# Do attention deficit/hyperactivity disorder symptoms exist among Brazilian indigenous children?

**DOI:** 10.1590/S1980-57642009DN30100008

**Published:** 2009

**Authors:** Paulo Verlaine Borges e Azevêdo, Leonardo Ferreira Caixeta

**Affiliations:** 1 School of Medicine, Universidade Católica de Goiás, Goiânia, GO, Brasil.; 2 School of Medicine, Universidade Federal de Goiás, Goiânia, GO, Brasil.

**Keywords:** Attention Deficit Disorder with Hyperactivity, mental health, child psychiatry, indigenous health

## Abstract

The validity of the clinical constructs of attention deficit hyperactivity disorder (ADHD) has been generalized for all human populations of different cultures worldwide. Yet important population groups have yet to be adequately studied so as to definitively confirm this generalization.

**Objective:**

To investigate the presence of symptoms of ADHD in children living within an indigenous community.

**Methods:**

We performed interviews in a bid to screen for symptoms of ADHD among settlement-dwelling indigenous children of the Karajá ethnic group in the Brazilian Amazonian Region.

**Results:**

Three narratives are presented highlighting the classical symptomatological triad of ADHD: inattentiveness, hyperactivity and impulsiveness.

**Conclusions:**

Some of ADHD’s major characteristics, described in the most common disease classification worldwide, are clearly described by children of this population. We concluded that ADHD symptoms are present in this population which diverges greatly in cultural issues compared to the Western world.

## Abstract (pt)

A validade dos construtos clínicos do transtorno de déficit de atenção e hiperatividade (TDAH) tem sido generalizados para todas as populações humanas de diferentes culturas pelo mundo afora. Ainda importantes grupos populacionais continuam exigindo estudos adequados de forma a confirmar definitivamente tal generalização.

**Objetivo:**

Investigar a presença de sintomas do TDAH entre crianças de comunidade indígena.

**Métodos:**

Realizamos entrevistas nas quais se explora a possibilidade da existência de sintomas do TDAH entre crianças indígenas da etnia Karajá, aldeiadas, na região da Amazônia Brasileira.

**Resultados:**

Três narrativas são apresentadas e evidenciam a tríade sintomatológica clássica do TDAH: desatenção, hiperatividade e impulsividade.

**Conclusões:**

Alguns dos sintomas característicos do TDAH, descritos nas classificações de doenças mais utilizadas no mundo, são claramente referidos por crianças dessa população. Concluímos que existem sintomas do TDAH nessa população, tão distinta culturalmente do mundo ocidental.

Approximately 4% to 5% of the world’s population is made up by indigenous peoples (approximately 250 million individuals). According to a WHO report, socio-economic-cultural aspects cannot be dissociated from any mental disorder without running the risk of envisaging conditions which do not actually exist within this type of human population.^1,2^ Our objective was to describe the presence of symptoms of ADHD in this specific population of youngsters aged no older than 16 years through collecting spontaneous reports by family members concerning their most frequent concerns. Subsequently, based on the gathered data from spontaneous reports, we evaluated whether there were presentations suggestive of what is known by other populations worldwide as symptoms of the clinical construct of attention deficit hyperactivity disorder (ADHD),^3-8^ a condition that may be questioned as simply having been imported from Western populations, and thus bearing no validity among other groups such as indigenous peoples.^1,2^

The Karajá Indians represent a rare group of natives who still live in settlements which are in relative isolation from Western culture.

## Methods

We conducted in-depth clinical and phenomenological interviews in 53 children of the Karajá ethnic group from Bananal Island, the planet’s largest fluvial island, located in the Legal Amazon region of Brazil. From an estimated population of 457 Karajá children aged 7–16 years living in the selected five settlements, 53 children (one child per family) representing 11.6% of children in this age range, were included in the study. These individuals represented all subjects that visited the health service over a period of one month (July, 2005) seeking assistance for mental complaints. Of the total of 53 children evaluated (32 males and 21 females), 13 presented symptoms and behaviors compatible with the construct of ADHD. Both parents and teachers were interviewed to check information on impairment.

The reports were produced using the Indian language through the help of health workers in charge of translation, as well as the Karajá Indians. The interviews were preceded by explanations such as, ‘Tell us about situations related to the behavior(s) of your child/children which are creating concern for the family, school, community and the child him/herself because we could seek, within the bounds of available resources, aid for their improvement, since a large number of these problems might have an associated medical component and as such, the use of medication and or psychological interventions could be important in relieving the problems’. Open-ended questions were, ‘Does this behaviour occur very frequently or sporadically?’ ‘Does it affect academic performance?’ ‘Does it affect personal and social relations?’ ‘Do they occur in more than one environment (home and school, for example)?’ ‘Did they start when the child was very young (under 7 years of age)?’ ‘Do they present additional behavioural problems?’ ‘What is the family pathological background?’ ‘What are the child’s neurological, psychological and motor conditions like?’.

The present research was approved by the Ethics Committee of the Universidade Federal de Goiás (UFG) and by the Brazilian Foundation for the Protection of Indigenous Populations (FUNAI). Parental informed consent was obtained before each interview. Children with symptoms suggestive of mental disorders were referred for specialized medical-psychological treatment.

## Results

From the 13 cases that presented symptoms and behaviors coherent with the construct of ADHD, three narratives are presented below to illustrate the reports which are suggestive of the presence of symptoms of ADHD.

### Case 1: 7 year-old boy

The mother reported that since the first year of life, the child has been quite restless, disobedient and does not seem to listen when talked to. He shows no respect for anybody and cannot sit still at school for a moment and is also a “slow learner” leading to several complaints regarding his poor learning compared to his classmates. He blinks excessively and has restless sleep and reduced appetite. He was born after a full 9-month problem-free pregnancy.

His Newborn Screening Test and vaccination were normal. He was able to walk at 1.5 years of age and to talk at the age of two. He presented no cranial-encephalic traumas, CNS infections, epileptic seizures or any other systemic medical problems. In the child’s mother’s words, he is intelligent and affectionate. She is a heavy smoker who smoked 20 cigarettes a day during the pregnancy.

### Case 2: 9 year-old girl

The mother reports that the child is far more restless and inattentive than her sister and the other children in the settlement. She is predominantly uninterested in school, and has difficulty learning. The mother described her as an intelligent and affectionate child. At school she “does not listen” to what her teachers say, keeps leaving the classroom and goes to the toilet “too often”. She sleeps well, but has poor appetite. She was a full term infant from a normal problem-free home delivery. She was breast fed up to the age of 2. She has normal vaccination. She was able to walk and speak at the age of 2. She presented no cranial-encephalic traumas, CNS infections, epileptic seizures or any other systemic medical problem. The mother used to abuse alcohol when single, is depressed most of the time and has always been “distracted” and had similar difficulties learning at school as her daughter. The father abuses alcohol and cigarettes and has used illegal substances (marijuana), is very irritable and physically and verbally assaults the child’s mother. Several of his relatives also have alcohol and cigarette-related problems.

### Case 3: 11 year-old boy

The child’s father reports that he has always been the ‘most restless’, who is ‘unable to wait for his turn’ and who ‘answers without thinking first’ and would always ‘meddle in other children’s play’. He has always been ‘inattentive and spaced out’. At the age of five, as a direct result of being so agitated, he was involved in an accident in which ‘a brick wall fell on him’. Since then, he has become even more agitated, edgy and aggressive towards other children and animals alike. ‘He is unable to learn anything at school’. He presented no cranial-encephalic traumas, CNS infections, epileptic seizures or any other systemic medical problem. He was able to walk at the normal age and to talk just at the age of 3.5 years. The father is an alcoholic, a smoker and ‘very irritable’, also being very restless and ‘finding it difficult to pay attention’.

## Discussion

Attention Deficit Hyperactivity Disorder (ADHD) has a prevalence of between 3% and 6% in different cultures.^3-8^ Studies among indigenous Indians are scarce, while studies among settlement-dwelling indigenous Indians are non-existent.

Epidemiological investigations into mental disorders and behavioural problems within Indigenous populations are scarce and when available present methodological problems which give rise to questions regarding their accuracy.^1,2^

The anthropological component becomes vital as it tests the validity of Western diagnostic categories concerning diverse cultures under study^1,2^. This study described findings of detailed clinical interviews performed in three native children that complain of symptoms of ADHD. Most notably, the classical symptomatological triad of ADHD, namely inattentiveness, hyperactivity and impulsiveness^3-8^ was spontaneously reported by the families, as was compromised performance amid family and school settings. We do not agree with the hypothesis put forth by Baydala et al.^9^ which claims that the unique behavioural and learning characteristics among indigenous children could mistakenly lead to the diagnosis, given their siblings and schoolmates present differentiated behaviour. Moreover, there is also the perception of the existence of a disorder as reported by individuals belonging to the community (mothers and teachers). We believe that these observations point to transcultural validation of at least the cardinal symptoms associated with the diagnostic category of ADHD.^3-8^ We suggest further research involving this population in order to elucidate the question over the presence or otherwise of ADHD, a disease detected among non-indigenous children and adolescents worldwide and in Canadian indigenous peoples.
